# Revisiting Hematoma Rates In Male Patients After Facelift Surgery: A Matched Cohort Analysis

**DOI:** 10.1093/asjof/ojag138.014

**Published:** 2026-07-24

**Authors:** Neel Vishwanath, Max Mandelbaum, Pierce L Janssen, Elad Fraiman, Viren Patel, Nicholas R Sinclair, James E Zins

**Affiliations:** Plastic Surgery Resident Department of Plastic Surgery, Cleveland Clinic Foundation, Cleveland, OH, USA; Aesthetic Plastic Surgery Fellow Department of Plastic Surgery, Cleveland Clinic Foundation, Cleveland, OH, USA; Aesthetic Plastic Surgery Fellow Department of Plastic Surgery, Cleveland Clinic Foundation, Cleveland, OH, USA; Medical Student Case Western Reserve University School of Medicine, Cleveland, OH, USA Research Fellow, Department of Plastic Surgery, Cleveland Clinic Foundation, Cleveland, OH, USA; Plastic Surgery Resident Department of Plastic Surgery, Cleveland Clinic Foundation, Cleveland, OH, USA; Plastic Surgery Attending Physician Department of Plastic Surgery, Cleveland Clinic Foundation, Cleveland, OH, USA; Emeritus Chair of the Department of Plastic Surgery & Section Head of Cosmetic Surgery Department of Plastic Surgery, Cleveland Clinic Foundation, Cleveland, OH, USA

## Abstract

**Goals/Purpose:**

Historically, male facelift patients have demonstrated higher postoperative hematoma rates than females, attributed to thicker skin, greater vascularity, elevated blood pressure, and lifestyle factors such as alcohol or tobacco use. However, with contemporary protocols and adjuncts, these disparities may be narrowing. This study evaluates postoperative outcomes between matched male and female facelift patients treated by a single surgeon using a standardized hematoma-prevention protocol. We hypothesized that modern perioperative strategies mitigate sex-based differences in hematoma rates.

**Methods/Technique:**

A retrospective matched cohort analysis was conducted of consecutive patients undergoing facelift surgery between January 2014 and January 2024 by a single senior surgeon. Male patients were compared with a propensity matched cohort of female patients, matched on age, body mass index (BMI), primary versus revision status, local tranexamic acid (TXA) use, smoking status, diabetes mellitus, and hypertension.

All procedures were performed under general anesthesia using either extended superficial musculoaponeurotic system (SMAS) or SMAS plication techniques, with standardized dissection, meticulous hemostasis, and postoperative blood pressure control. Local infiltration included 0.5% lidocaine with 1:200,000 epinephrine; beginning in 2019, TXA (1–2 mg/mL) was added to the tumescent mixture. The second-look technique was employed for all facelifts, which entails delayed hemostasis and closure of one side before proceeding to the other to allow epinephrine’s vasoconstrictive effects to subside and delayed bleeding to appear. The hemostatic net was adopted in 2023 for all male patients and high-risk females. Systolic blood pressure was maintained <140 mm Hg intra- and postoperatively with intravenous labetalol and hydralazine. Closed-suction drains were used in all cases.

Demographics, comorbidities, operative variables (technique, adjuncts, operative time, estimated blood loss [EBL]), and outcomes were recorded. Minor complications were those managed nonoperatively (hematoma/seroma aspirated in the office or with local I&D, neuropraxia 3 months, or chronic wound/major necrosis needing revision). Caliper-based propensity matching was applied. Continuous variables were compared with t-test or Mann– Whitney U, categorical variables with χ² or Fisher’s exact test, with significance set at P < 0.05.

**Results/Complications:**

Sixty-eight patients met inclusion criteria: 34 male and 34 female. The mean age was 66.4 years (SD 0.8), with no difference between groups (66.5 vs 66.2 years, P=0.73). BMI was similar (26.2 kg/m² vs 26.2 kg/m², P=0.31). Rates of hypertension (38.2% vs 41.1%, P=0.80), diabetes (5.9% both, P=1.0), and secondary facelift status (11.8% vs 20.6%, P=0.32) were comparable. Two patients were active smokers, both male (5.9% vs 0%, P=0.49). Median follow-up was 302 days [IQR 76–537].

Most (83.9%) underwent extended SMAS facelift (80.6% vs. 87.1%, P=0.49). TXA use was comparable (50% vs. 53%, P=0.81). Male patients demonstrated greater intraoperative blood loss >100 cc (34.8% vs. 6.9%, P=0.02) and longer operative time (457 [IQR 435–491] vs. 422 [IQR 375–439] min, P<0.001). Fat grafting was more frequent in females (67.6% vs. 29.4%, P=0.01), while other adjuncts (blepharoplasty, platysmaplasty, chemical peel) were similar.

Despite increased operative time and blood loss in males, no difference in postoperative hematoma rate was observed (0% vs. 2.9%, P = 1). No hematomas required operative evacuation. The overall hematoma incidence (1.5%) was substantially below historical benchmarks, reflecting the cumulative effect of modern hematoma prevention strategies.

Remaining postoperative outcomes and total complication rates were comparable. No major complications occurred in males; one female was readmitted for infection (0% vs. 2.9%, P = 1). There were no wound-healing complications requiring readmission, permanent facial-nerve injuries, or other major adverse events (all 0%, P=1). Minor issues—transient neuropraxia (0% males vs. 5.9% females, P=0.49) and superficial infection (0% males vs. 8.8% females , P=0.24)—were rare and not statistically significant.

**Conclusion:**

This matched cohort study demonstrates that, within a facelift protocol emphasizing strict blood pressure control, the second look technique, adjunct use of the hemostatic net, and local TXA infiltration, hematoma rates are dramatically reduced across both sexes. As male patients historically experienced higher baseline hematoma risk, these advances appear to have proportionally benefited them, resulting in hematoma rates comparable to females. In turn, while male facelifts exhibited greater intraoperative bleeding, these differences did not translate to higher rates of hematoma, reoperation, or overall complications.

Rather than implying the disappearance of biologic differences, these results highlight the progress achieved through standardized perioperative management and technical refinement. The results support the effectiveness of contemporary hematoma prevention strategies and underscore the safety of facelift surgery in male patients when performed under rigorous modern protocols.

Overall, this contributes to a growing body of literature suggesting that contemporary facelift techniques increasingly emphasize safety—particularly in addressing hematoma, a complication that has historically plagued male patients at higher rates.

